# Clinical experience of biliary T tube of immobilization of peri-pin membrane in tibial Gustilo III fracture treated with vacuum sealant drainage combined with an external fixator

**DOI:** 10.1097/MD.0000000000022846

**Published:** 2020-10-23

**Authors:** Hui Ye, Shufeng Lin, Junfeng Zhu, Lifeng Jiang

**Affiliations:** a Department of Orthopedics Surgery, Suichang branch of The Second Affiliated Hospital, Zhejiang University School of Medicine (Suichang County People's Hospital in Zhejiang Province), Suichang, LiShui; b Department of Orthopedics Surgery, The Second Affiliated Hospital, Zhejiang University School of Medicine, Hangzhou, China.

**Keywords:** air leakage, external fixation, gustilo III type, T-tube, vacuum sealant drainage

## Abstract

**Objective:**

To determine the effects of an improved method of peri-pin membrane immobilization in tibial Gustilo type III fracture treated with vacuum sealant drainage (VSD) combined with an external fixator.

**Method:**

A biliary T tube of suitable size and type was cut into a certain long arm and cross arm which wrapping around a pin to improving traditional peri-pin VSD method. Eighty-six cases of Gustilo type III tibial fracture admitted from January 2016 to December 2019 were prospectively treated, of which 43 cases were treated using a traditional method of VSD (Traditional group) and 43 cases in which VSD treatment was enhanced (Improved group). The 2 groups were compared by some clinical indexes. Statistical software was then used for data analysis. *P* < .05 was considered statistically significant.

**Results:**

Compared with the Traditional group, the improved group significantly reduced granulation tissue growth time (day) (7.35 + 2.59 vs 11.14 + 2.54, *P* < .05), antibiotic use time (day) (6.67 + 2.39 vs 8.70 + 1.98, *P* < .05), operation time (min) (72.44 + 16.79 vs 85.47 + 17.44, *P* < .05) duration of hospital stay (day) (18.23 + 5.04 vs 21.53 + 4.79, *P* < .05), wound closure time (day) (9.23 + 2.69 vs 14.19 + 2.67, *P* < .05), air leakage around the fixed needle (3/43 vs 16/43, *P* < .05) and postoperative pain score (*P* < .05). Meanwhile, the white blood cell, C-reactive protein, erythrocyte sedimentation rate of 1 week and 2 weeks post-operation were also reduced after adopting the improved method (*P* < .05). The difference in infection around the fixation pin and pin loosening between the 2 groups was not significant.

**Conclusion:**

The biliary T tube was effctive in improving VSD combined with external fixation for the treatment of tibial Gustilo type III fractures. The materials are easy to obtain and straightforward to use and so is worthy of clinical promotion.

## 1 Introduction

Tibial Gustilo type III fractures are mostly caused by high-energy injuries, often with large-scale skin contusions and defects, resulting in exposure of the bone and wound contamination.^[1]^ The management of open bone injuries combined with soft tissue defects remains a difficult problem in clinic.^[2]^ At present, vacuum sealant drainage (VSD) combined with external fixator technology is widely used in clinical treatment, with which satisfactory results have been obtained.^[3]^ VSD can accelerate the discharge of purulent secretions, promote wound healing and promotion of granulation tissue growth.^[4–6]^ External fixation pin is not only flexible, portable, but also simple, safe, and effective. It causes little damage to soft tissues, does not increase periosteal detachment or soft tissue injury.^[7]^ VSD combined with an external fixation pin for the treatment of tibial Gustilo type III fractures is effective in preventing infection, promoting wound healing, and maintenance of relative stability of the fractured bone.^[8,9]^ However, due to the combined use of VSD and external fixation pin, the VSD dressing is often found to be in contact with the external fixation pin. The surgeon experiencing difficulty in ensuring negative pressure is achieved around fixation pins during surgery, leading to air leakage post-surgery which affects treatment outcomes.^[10]^ The air leakage prevents the VSD completely removing secretions and necrotic tissue in the cavity and wound surface, increasing the possibility of wound infection. The phenomenon of air leakage is often experienced following surgery.^[11]^ Air leakage can accelerate the formation of dry wound secretions, condensation blocking the drainage tube, resulting in further reduction of drainage tube pressure, leading to a vicious cycle.^[3]^

In addition, in such open fractures, multiple VSD dressings are required for large-scale wounds. There are multiple drainage tubes and a large area to drain.^[12]^ The relative negative pressure in each drainage tube is low, and the flow rate of the drainage liquid slow. Secretions are more likely to dry out or accumulate after air leakage. Sun et^[3]^ reported that a rubber strip is wrapped around each pin in 3 circles outside the plastic drape could resloved the air leakage. However, we think that this approach is still flawed, because the strip is on the outside of the membrane. In view of the difficulty of ensuring negative pressure around fixation pins and the problem of air leakage following surgery, we improved the method of sealing around the VSD and fixation pins.

## 2 Material and methods

### 2.1 General information

A prospective study was performed after obtaining institutional review board approval following the Declaration of Helsinki principles. From January 2016 to December 2019, 86 cases of tibial Gustilo type III tibia and fibula fractures were selected by the Orthopedics Department for treatment with VSD combined with external fixation pins, with at least 1 fixation pin in the area covered by the VSD dressing. A total of 43 cases of VSD were closed using a modified method (Improved group), and 43 cases using the traditional method (Traditional group). The Improved group consisted of 28 males and 15 females, the mean age of whom was 44.72 + 12.14 years old. The Traditional group consisted of 31 males and 13 females, the mean age of whom were 42.95 + 10.80 years old. A comparison of the general data between the 2 groups demonstrated that there was no statistically significant difference in age, gender, or BMI (*P* > .05)(Table 1).

**Table 1:**
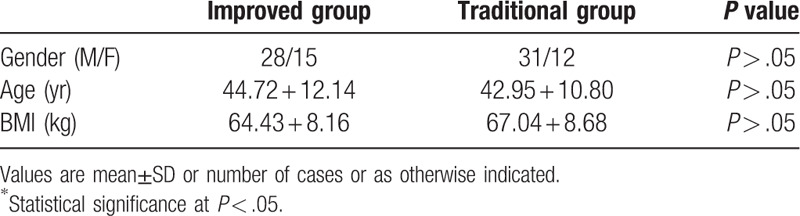
Comparison of preoperative clinical data between improved group and traditional group.

### 2.2 Therapeutic methods

**Figure 1:**
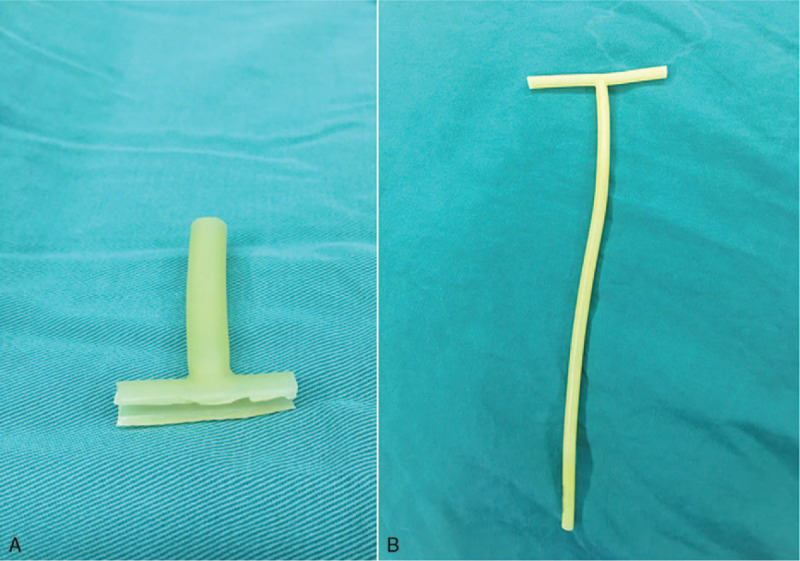
A suitable size and type of bile duct T-tube whose diameter was slightly smaller than that of the fixation pin was selected, then was cut a length of approximately 5 cm of the long arm and a length of approximately 1 cm of the cross arm, and the horizontal The bottom of the cross arm was cut and turned outward to form a flat portion.

**Figure 2:**
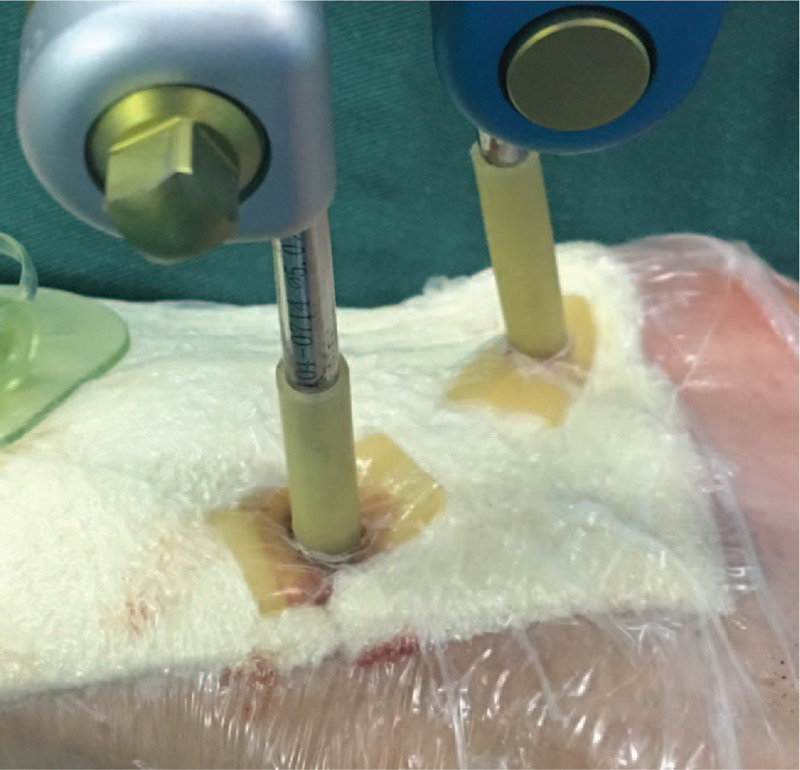
The pin sleeve was placed into the long arm of the T tube. The bottom of each cross arm was cut open and turned outwards to form a flat section, placed over the VSD surface, and the film was placed over the T tube and VSD surface to form a seal. VSD = vacuum sealant drainage.

**Figure 3:**
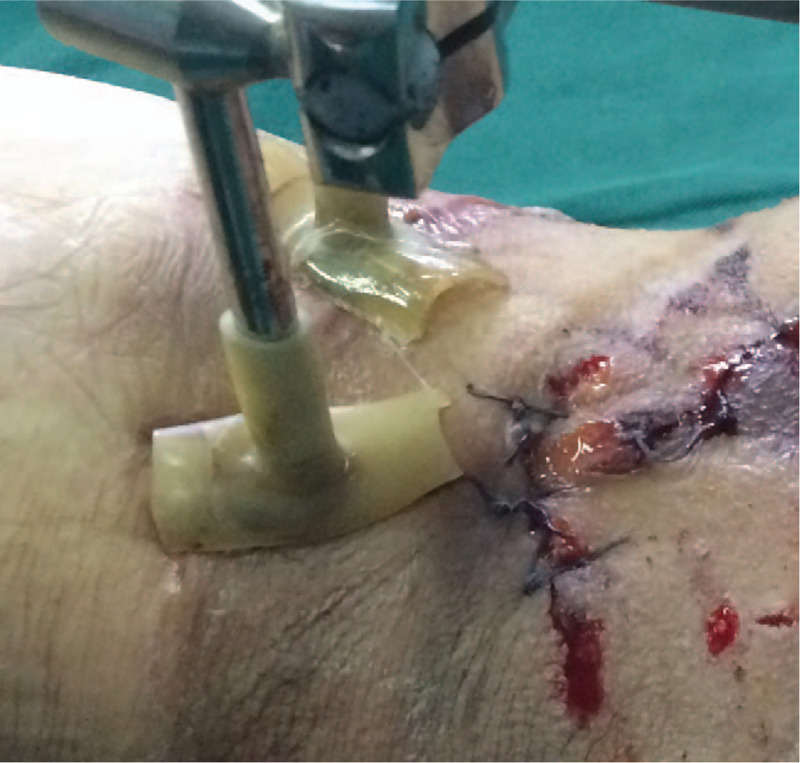
After removing the VSD, the position relationship between the trimmed t-tube and the fixed needle which can be used for multiple times after cleaning and disinfection. VSD = vacuum sealant drainage.

During surgery, complete debridement was initially performed, with removal of severely contaminated soft tissues, large and small bone fragments, with the surrounding soft tissues retained as far as possible to avoid damaging blood circulation. A unilateral external stent was installed after reduction of the fractured ends. Damaged blood vessels, nerves and tendons were then reconstructed, after which the wound was washed repeatedly with normal saline and hydrogen peroxide until the wound was clean. A 1% iodophor wet compress was applied for 5 to 10 minutes, after which the wound was washed again with normal saline. A suitable VSD was cut to cover or fill the wound surface which was then sutured to the surrounding skin.^[13]^ In the Improved group, a biliary T tube of suitable size and type (the diameter of the T tube selected was slightly smaller than that of the fixation pin) was cut such that the long arm had a length of approximately 5 cm, and the 2 sides of the cross arm having a length of approximately 1 cm (Fig. 1). The pin sleeve was placed into the long arm of the T tube. The bottom of each cross arm was cut open and turned outwards to form a flat section. The film was placed over the T tube to form a seal. In the Traditional group, the film was trimmed into strips. The guide pin was covered by the VSD in the form of a horizontal and vertical cross (using a “tile-laying method”) (Figs. 2 and 3). A negative pressure suction device was attached and the clinical effects recorded.

### 2.3 Postoperative treatment

For the first 24 hours of continuous drainage, negative pressure was set to 50 ∼ 70 kPa. The patient's general condition was observed, especially local wound drainage and peripheral circulation of the limbs. Vital signs were recorded, in addition to drainage volume and peripheral blood oxygen saturation. The use of antibiotics was selected based on bacterial culture and drug sensitivity tests. If the VSD continued to be effective, it was removed after 5 days of negative pressure. It was replaced at any point if it had failed. Depending on the wound situation, VSD treatment was continued, otherwise, when the wound surface is clean and the granulation grows fresh, the skin grafting and corresponding flap was created to cover the wound if this was required.

### 2.4 Observed indicators

Observation and treatment of postoperative rehabilitation indicators (granulation growth time, duration of antibiotic use, duration of surgery, length of hospitalization stay, wound closure time), preoperative and postoperative pain visual analog scale (VAS) scores, blood inflammation indicators, and incidence of postoperative adverse events (fixed), leakage or infection around the fixation pins or fixator loosening were used as the principal indicators to compare treatment effects between the Improved and Traditional groups.

### 2.5 Statistical methods

A database was established using an Excel spreadsheet that was used to enter and record survey data. The data were processed using SPSS 17.0 statistical software. Measurement data were expressed as means ± standard deviation (x ± s). A t-test was used for comparison between groups. A *χ*^2^ test was used for rate comparisons. *P* < .05 was considered statistically significant.

## 3 Results

### 3.1 The application of biliary T tube can significantly shorten the time of wound recovery and other related indicators

Table 2 shows that granulation growth time was siginificantly shorter in Improved group compared with the Traditional group (7.35 + 2.59 vs 11.14 + 2.54 days, *P* < .05). Also antibiotic use time was shorter in Improved group (6.67 + 2.39 days) than the Traditional group (8.70 + 1.98 days, *P* < .05). The operation time was shorter in Improved group than that of the Traditional group (72.44 + 16.79 vs 85.47 + 17.44 mins, *P* < .05). Compared with the Traditional group, the DHS (days) and wound closure time (day) in the Improved group were also siginificantly reduced, (18.23 + 5.04 vs 21.53 + 4.79 days, *P* < .05, 9.23 + 2.69 vs 14.19 + 2.67 days, *P* < .05), respectively.

**Table 2:**
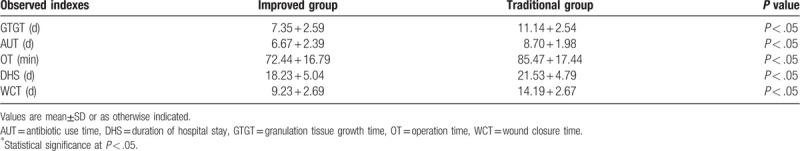
Comparison of time data related to treatment between improved group and traditional group.

### 3.2 The application of biliary T tube can significantly relieve postoperative pain

Pain in the preoperative wound area of the 2 groups was not significantly different. The pain in the 2 groups at 1 day, 3 days, 1 week and 2 weeks postoperation were less than that prior to surgery. However, the VAS scores in the Improved group was significant lower compared with the Traditional group 1st day PPS (6.67 + 1.25, vs 7.44 + 1.14, *P* < .05), 3 days PPS (5.28 + 0.93 vs 6.56 + 1.14, *P* < .05), 1 week PPS (3.42 + 1.14 vs 4.74 + 1.12, *P* < .05) and 2 weeks PPS (2.14 + 0.86 vs 2.81 + 0.96, *P* < .05), respectively (Table 3).

**Table 3:**
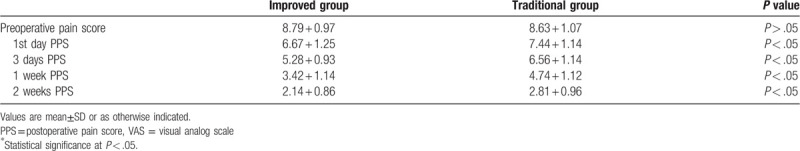
Comparison of VAS scores of pre- and post-operative pain between improved group and traditional group.

### 3.3 The application of biliary T tube can significantly reduce inflammatory markers

As show in Table 4, prior to treatment, inflammation indicators of the 2 groups were no significantly different. After treatment, the white blood cell (WBC), erythrocyte sedimentation rate (ESR), and C-reactive protein (CRP) levels of both group of patients were significantly lower. There was no statistical difference between the 2 groups first day postopration. However, the WBC, ESR, and CRP levels 1 week postoperation significantly reduced in the Improved group, comparing with the Traditional group. (8.16 + 1.45 vs 9.67 + 1.59, *P* < .05, 44.16 + 8.89 vs 44.16 + 8.89, *P* < .05, 46.73 + 8.60 vs 60.00 + 11.62, *P* < .05, respectively). The WBC, ESR, and CRP levels 2 weeks postoperation were also lower in the Improved group than in the Traditional group. (5.88 + 1.48 vs 8.05 + 1.44, *P* < .05, 29.98 + 5.81 vs 34.44 + 5.32, *P* < .05, 9.56 + 2.93 vs 14.37 + 6.02, *P* < .05, respectively).

**Table 4:**
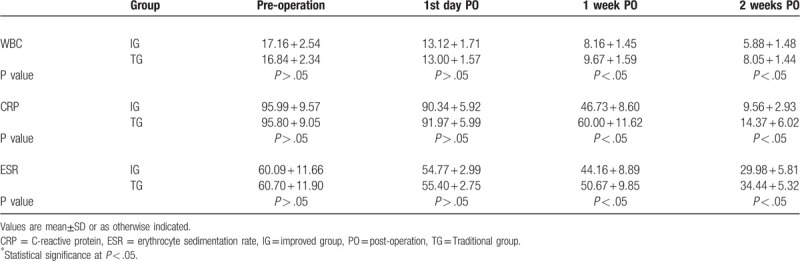
Comparison of inflammation indexes between improved group and traditional group of patients before and after treatment.

### 3.4 The application of biliary T tube can significantly reduce the air leakage rate

Compared with the traditional group, air leakage around the fixation pins of the Improved group was significantly lower (3/43 vs 3/43, *P* < .05). However, there was no significant differences in infection around the fixation pin (1/43 vs 3/43, *P* > .05) and pin loosening (2/43 vs 5/43, *P* > .05) between the 2 groups (Table 5).

**Table 5:**
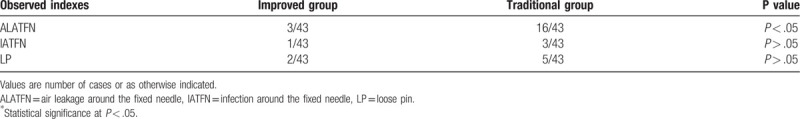
Comparison of the incidence of postoperative adverse events between improved group and traditional group.

## 4 Discussion

Clinical treatment of Gustilo type III tibial fractures is relatively difficult.^[14]^ In recent years, the majority of clinicians have used external fixation pins combined with VSD technology to treatment, and have achieved satisfactory results.^[9]^ However, the VSD dressing interface may need to cover the external fixation pin, making it difficult to seal the membrane around the fixation pin, air is often leak after surgery.^[15]^ Some researchers have suggested that the external fixation pin should be completely sealed,^[16]^ but this was complicated and increases the cost to the patient. Other researchers have advocated the use of hydrophilic gel around the pins, a solution that has been commercialized. However, these methods still cannot completely solve the problem of VSD air leakage around the external fixation pins.

The improved method used in the present study are similar to the “valve core” principle. A surgical T tube of an appropriate size (with an inner diameter slightly smaller than the outer diameter of the fixation pins) was selected. The rubber T-tube was trimmed and placed on the fixator, with the film covering the upper surface of the bottom of the T tube in order to form a closed interface. Only a single layer of film was required to cover the wound, which is economical and convenient.

VSD combined with external fixation has been reported in previous studies and good results have been obtained.^[8,15,17]^ However, little attention has been paid to the improvement of VSD leakage. The improved method effectively maintained negative pressure in the VSD and continued to be effective in maintaining blood flow through the wound following surgery. This has the consequence of enhancing the repair, significantly shortening the time for granulation tissue, the duration of antibiotic use and the time required for complete wound closure. In addition, the length of hospital stay was also reduced. The results of this study demonstrated that the VAS pain scores and inflammatory indicators of patients in the modified group following treatment were significantly lower than those in the traditional group. The most important reason to reduce these indicators is that the improved method could significantly reduced the incidence of air leakage around the fixation pins. However, there was no significant difference between the 2 groups (infection around the fixation pins and pin loosening). The author thinks that this may be due to the fact that the incidence of infection and loosening around the external fixation pin was not high. However, while the 2 groups were not significantly different, the improved group demonstrated a lower trend of problems.

The improved method using T-tube combined with VSD and an external fixation pin for the treatment of tibial Gustilo type III fracture was an effective method. The method is simple and cheap, so is worthy of clinical promotion.

## Acknowledgments

We are very grateful to the patients for participating in the study. Thanks to the team for their efforts.

## Author contributions

**Data curation:** Hui Ye.

**Formal analysis:** Lifeng Jiang.

**Investigation:** Hui Ye, Shufeng Lin.

**Methodology:** Hui Ye.

**Project administration:** Shufeng Lin.

**Resources:** Hui Ye, Shufeng Lin.

**Software:** Lifeng Jiang.

**Supervision:** Lifeng Jiang.

**Validation:** Lifeng Jiang.

**Visualization:** Shufeng Lin, Junfeng Zhu.

**Writing – original draft:** Lifeng Jiang.

**Writing – review & editing:** Junfeng Zhu.
